# Male Infertility Coexists with Decreased Sperm Genomic Integrity and Oxidative Stress in Semen Irrespective of Leukocytospermia

**DOI:** 10.3390/antiox11101987

**Published:** 2022-10-05

**Authors:** Kamil Gill, Tomasz Machalowski, Patryk Harasny, Michal Kups, Marta Grabowska, Ewa Duchnik, Olimpia Sipak, Monika Fraczek, Maciej Kurpisz, Rafal Kurzawa, Malgorzata Piasecka

**Affiliations:** 1 Department of Histology and Developmental Biology, Faculty of Health Sciences, Pomeranian Medical University, 71-210 Szczecin, Poland; 2 Department of Perinatology, Obstetrics and Gynecology, Faculty of Medicine and Dentistry, Pomeranian Medical University, 72-010 Police, Poland; 3 Department of Urology and Urological Oncology, Faculty of Medicine and Dentistry, Pomeranian Medical University in Szczecin, 70-111 Szczecin, Poland; 4 Department of Urology and Oncological Urology, Regional Specialist Hospital in Szczecin, 71-455 Szczecin, Poland; 5 The Fertility Partnership Vitrolive in Szczecin, 70-483 Szczecin, Poland; 6 Department of Aesthetic Dermatology, Faculty of Health Sciences, Pomeranian Medical University in Szczecin, 70-111 Szczecin, Poland; 7 Department of Obstetrics and Pathology of Pregnancy, Faculty of Health Sciences, Pomeranian Medical University in Szczecin, 71-210 Szczecin, Poland; 8 Institute of Human Genetics, Polish Academy of Sciences, 60-479 Poznan, Poland; 9 Department of Gynecology and Reproductive Health, Faculty of Health Sciences, Pomeranian Medical University in Szczecin, 71-210 Szczecin, Poland

**Keywords:** male infertility, semen, leukocytospermia, sperm DNA fragmentation, oxidation-reduction potential in semen, oxidative stress

## Abstract

Our research was designed to verify the relationship between male infertility, basic semen characteristics (with respect to detailed sperm morphology), sperm DNA fragmentation (SDF), oxidation-reduction potential in semen (ORP), and leukocytospermia. The obtained results showed that infertile groups (with or without leukocytospermia) had significantly lower basic semen characteristics and higher SDF, raw ORP, and static ORP (sORP) than fertile controls. The thresholds of 13% SDF (AUC = 0.733) and 1.40 sORP (AUC = 0.857) were predictive values for discriminating infertile from fertile men. In infertile groups, a higher prevalence and risk for >13% SDF and >1.40 sORP were revealed. Unexpectedly, leukocytospermic subjects had lower sORP, prevalence, and risk for >1.40 sORP than leukocytospermic-negative men. These groups did not differ in SDF and raw ORP. Both SDF and sORP negatively correlated with basic semen parameters but positively correlated with sperm head and midpiece defects. sORP positively correlated with sperm tail defects, immature sperm cells with excess residual cytoplasm, and SDF. In turn, raw ORP negatively correlated with sperm count but positively correlated with SDF and sORP. These findings indicate that (1) there is a relationship between male infertility, SDF, and OS in semen; (2) in infertile men, there is a clinically significant risk of SDF and OS irrespective of leukocytospermia; and (3) the assessment of SDF and oxidative stress should be independent of leukocytospermia.

## 1. Introduction

Infertility affects 10–20% of couples worldwide. This has become a social and civilization issue and poses a serious challenge for public health. On the basis of recent epidemiological data, the male factor (coexisting with a female factor) contributes to 20–70% of these issues, depending on the latitude. In central Europe, the male factor is responsible for approximately 60% of couples’ infertility cases. The etiopathogenesis is complex and multifactorial (e.g., genetic, hormonal, immunological, inflammatory/infectious factors, varicocele, systemic disease, lifestyle, environmental pollution) and often difficult to identify. Idiopathic infertility (i.e., an unexplained decrease in semen quality with no history associated with fertility problems and normal findings on physical examination and endocrine laboratory testing) is found to a significant extent (30–50% of cases). In 30–80% of clinical trials, oxidative stress (OS) occurs in semen, which is considered to be one of the main causes of nuclear sperm DNA fragmentation. To describe OS-associated male infertility (men with abnormal sperm parameters and OS in semen), scientific researchers proposed the term ‘male oxidative stress infertility’ (MOSI) [1,2,3,4,5,6,7,8,9].

It is believed that male genital tract inflammatory processes and infections (e.g., sexually transmitted diseases, testicular injury, varicocele, epididymitis, prostatitis, urethritis) are significant etiological factors of OS and are diagnosed in approximately 15% of infertile men. In many cases, local inflammations/infections are asymptomatic and often coexist with an increased concentration of leukocytes (WBCs, white blood cells) in semen and with an imbalance between oxidative and antioxidative processes manifested by an increase in reactive oxygen species (ROS), consequently leading to an abnormal oxidation-reduction potential (ORP) in semen [2,6,9,10,11,12,13,14,15].

According to the WHO (World Health Organization), if the concentration of peroxidase-positive leukocytes is ≥1 × 10^6^/mL ejaculate, it is defined as leukocytospermia (pyospermia). Many clinical trials have revealed that up to 30% of infertile men suffer from this condition. Seminal plasma WBCs are mainly phagocytic cells, such as polymorphonuclear granulocytes (PMNs) (accounting for 50–60% of WBCs) and macrophages (accounting for 20–30% of WBCs). Moreover, lymphocytes (2–3%) were also noted in seminal plasma WBCs. Under normal physiological conditions (WBCs < 1 × 10^6^/mL ejaculate), inflammatory cells play key roles in immunosurveillance and in removing abnormal sperm cells. Thus, these cells control the quality of semen and prevent damaged male gametes from entering the female reproductive system to facilitate successful fertilization. Moreover, leukocytes are involved in the production of ROS (up to 1000 times more than spermatozoa), which are essential (in small amounts) for important reproductive processes, such as capacitation, hyperactivation, acrosomal reaction, and sperm–oocyte fusion. ROS play the role of signaling molecules that promote the abovementioned processes. Active leukocytes are known to generate up to 100-fold more ROS than inactive leukocytes [6,10,11,16,17,18,19,20,21,22]. Therefore, these active cells are considered to be the primary source of ROS responsible for negatively affecting sperm cell quality (e.g., motility, morphology, vitality), especially the integrity of their nuclear and mitochondrial genome, which, as a consequence, may contribute to a reduction in sperm-fertilizing ability. Moreover, after stimulation by pathogens, WBCs also release proinflammatory cytokines (e.g., IL-6 and IL-8), proteases (e.g., cathepsin G, collagenase, and elastase), and chemotactic factors [12,16,23,24,25,26,27,28,29,30,31].

However, it should be emphasized that leukocytospermia (an endogenous source of ROS) is not the only cause of OS. Endogenous causes also include varicocele and immature forms of sperm cells (with excess cytoplasmic residues in the midpiece) appearing in semen as a result of spermiogenesis disorders (abnormal sperm morphogenesis—retention of residual cytoplasm of elongated spermatid). Sperm residual cytoplasm contains a key enzyme of the hexose monophosphate (HMP) shunt, glucose-6-phosphate dehydrogenase (G6PD), which controls the production of β-nicotinamide adenine dinucleotide phosphate (NADPH). This compound (a source of electrons) is used for the reduction of oxygen to produce the ROS superoxide anion. The transfer of electrons from NADPH to molecular oxygen is catalyzed by the membrane-bound enzyme NADPH oxidase (NOX). In addition, sperm mitochondria located in the midpiece are able to generate ROS (e.g., superoxide anion, hydroxyl radical, hydrogen peroxide) via complexes I and III of the electron transport chain (ETC) situated in the inner mitochondrial membrane. Dysfunction of the ETC leads to the release of pathological amounts of ROS. Oxido-reductases, cooperating with the ETC, play an important role in the generation of ROS [10,16,18,19,20,21,23,27,32,33,34]. It cannot be ignored that in the case of sperm morphogenesis disorders, when there is an abnormal rejection/elimination of the residual cytoplasm of the elongated spermatid, some of the mitochondria that have not been incorporated/recruited (redundant mitochondria) into the midpiece remain in the cytoplasm [35]. Thus, both the residual cytoplasm and its mitochondria can be a source of excess intrinsic ROS.

Currently, attention is also being paid to the exogenous causative agents of OS in the male genital tract. These causes include lifestyle factors (e.g., stress, alcohol, smoking, fatty diet, drug addictions), health conditions (e.g., testicular diseases, chronic diseases, medications), and environmental pollution (e.g., heavy metals, air pollution, pesticides, radiation) [6,9,18,19,20,21,27].

Because oxidative stress in semen results in low sperm quality and therefore in many cases contributes to adverse effects on male fertility and can be caused by different factors (endo- and exogenous), our research was designed to verify the relationship between male infertility, basic semen characteristics (with respect to detailed sperm morphology), sperm DNA fragmentation, oxidation-reduction potential in semen, and leukocytospermia.

## 2. Materials and Methods

### 2.1. Participants

For this study, 204 male participants (median age: 33.00 years; range: 24–49 years) were enrolled. In this population, three groups were designed: (1) 47 infertile men with leukocytospermia; (2) 77 infertile men without leukocytospermia; and (3) 80 nonleukocytospermic volunteers with proven fertility. All infertile patients met the World Health Organization (WHO) criteria of infertility (no pregnancy within at least one year of regular unprotected sexual intercourse) and were treated in 2021–2022 in the Individual Specialist Medical Practice (Szczecin, Poland) and TFP Vitrolive in Szczecin (Poland)—Gynecology and Fertility Clinic. To verify the infertile status of men, medical interviews and physical examinations (conducted by a specialist in urology—M. K.) were performed. The interview and examinations included: history of genital injuries/disorders (e.g., cryptorchidism, varicocele, urogenital infections, chronic diseases, operations, and treatments) and exposure to harmful factors (e.g., pharmacotherapy, use of anabolic steroids, unhealthy lifestyle, stimulants, and others). Moreover, body proportions, penis, scrotum, and accessory glands were assessed. The subjects with cryptozoospermia, azoospermia, a history of testicular torsion or atrophy, maldescent testis, systemic and endocrine disease, current radiochemotherapy, or exposure to gonadotoxins were excluded from this study. In turn, the control group consisted of men who had fathered a child (natural conception) in the two years preceding the study. Fertile volunteers declared a lack of any known reproductive hazards that could have occurred from the time of conception to the day of this study. This group was recruited based on information posted on social media. In line with the ethical standards and the Declaration of Helsinki, all enrolled men signed an informed consent for participation in this research project (this study was approved by The Ethics Committee of Pomeranian Medical University, Szczecin, Poland; ethical authorization number: KB-0012/03/2021). All subjects reported to the Laboratory of Andrology in the Department of Histology and Developmental Biology (Faculty of Health Sciences, Pomeranian Medical University in Szczecin, Poland) for semen analysis.

### 2.2. Basic Semen Analysis

Semen samples were analyzed according to WHO recommendations [36]. The samples were collected in a sterile urine container by masturbation after a 2–7-day sexual abstinence. After complete liquefaction of semen (at 37 °C), standard semen analysis was conducted at room temperature (22 °C). Initial macroscopic assessment of the samples included color, viscosity, volume, and pH. In the next step, microscopic assessment (DM500 light/phase-contrast microscope, Lecia, Heerbrugg, Switzerland) included verification of sperm aggregation and agglutination and evaluation of the sperm concentration and total sperm count, motility (progressive and nonprogressive motility), morphology, vitality, and concentration of round cells (round cells of spermatogenic lineage and inflammatory cells). The cell concentration (performed with the use of an improved Neubauer hemocytometer—Heinz Hernez Medizinalbedarf GmbH, Hamburg, Germany), sperm motility, and vitality (eosin-negative cells (eosin test) and hypoosmotic-reactive cells (HOS test)) were assessed under a light/phase-contrast microscope using a 40× objective. Eosin and HOS tests are complementary and reveal the integrity of the cellular membrane at the sperm head and flagellum, respectively. To verify the sperm cell morphology (including the teratozoospermia index, reflecting multiple morphological defects per spermatozoon—TZI), native sperm smears were fixed and stained according to the Papanicolaou method (Aqua-Med, Lodz, Poland) and were analyzed under a bright light microscope using a 100× objective oil immersion lens. Detailed sperm morphological analysis included sperm head defects, midpiece defects, tail defects, and immature sperm cells with residual cytoplasm [36]. To distinguish leukocytes (peroxidase-positive cells) from round cells of spermatogenic lineage, the Endtz test (LeucoScreen kit, FertiPro N.V., Beernem, Belgium) was used.

In the group of infertile leukocytospermic patients (≥10^6^ peroxidase-positive cells/mL), only 7 out of 47 semen samples were classified as normozoospermia (total sperm count ≥ 39 × 10^6^ cells, sperm progressive motility ≥ 32%, and normal sperm morphology ≥ 4%) while 5 were classified as asthenoteratozoospermia (simultaneously abnormal progressive sperm motility and morphology), 14 were classified as oligoasthenoteratozoospermia (simultaneously abnormal total sperm count, progressive motility and morphology), 3 were classified as oligoteratozoospermia (simultaneously abnormal total sperm count and morphology), and 18 were classified as teratozoospermia (abnormal sperm morphology). In the group of infertile nonleukocytospermic patients, the distribution of the semological categories was as follows: 4 out of 77 obtained semen samples were classified as normozoospermia, 7 as asthenoteratozoospermia, 2 as oligozoospermia (abnormal total sperm count), 29 as oligoasthenoteratozoospermia, 17 as oligoteratozoospermia, and 18 as teratozoospermia. In turn, in the group of fertile volunteers, the distribution was as follows: 40 out of 80 semen samples were classified as normozoospermia, 1 as asthenozoospermia (abnormal sperm motility), 1 as asthenoteratozoospermia, 5 as oligozoospermia, 1 as oligoasthenoteratozoospermia, 1 as oligoteratozoospermia, and 31 as teratozoospermia. 

### 2.3. Sperm Chromatin Dispersion (SCD) Test

Based on the chromatin dispersion method, the fragmentation of sperm nuclear DNA was verified. For this purpose, a diagnostic test—Halosperm G2^®^ kit (Halotech DNA, Madrid, Spain)—was applied. The laboratory procedure was conducted strictly according to the manufacturer’s guidelines. The specific steps of this procedure were described in detail in our previous publications [37,38,39,40,41,42].

To calculate the percentage of sperm cells with SDF, a minimum of 300 sperm cells per sample were counted under the 100x objective of a bright light microscope. The evaluation of sperm DNA integrity/fragmentation was performed according to the manufacturer’s guidelines (Table 1). The results of the SCD test (SDF) are presented as the percentage of sperm cells with damaged sperm nuclear DNA (sum of spermatozoa with nuclear DNA fragmentation divided by the total number of assessed sperm cells and multiplied by 100%). According to the manufacturer’s recommendations, >30% SDF was considered abnormal and related to a high risk of male infertility.

### 2.4. Static oxidation-reduction Potential (sORP) in Semen

The oxidation-reduction potential in semen was verified using the Male Infertility Oxidative System (MiOXSYS^®^, Aytu BioScience, Englewood, OH, USA). The measurements were performed in accordance with the manufacturer’s guidelines. After semen liquefaction, 30 μL of sample was dropped into the sample port of a disposable MiOXSYS^®^ sensor and inserted into a MiOXSYS analyzer. Measurements were performed only on fully liquefied samples with the correct viscosity. The raw ORP was measured in millivolts (mV). Fallowing the manufacturer’s protocol, the raw ORP value was normalized to the sperm concentration and represented as the static ORP (sORP, expressed as mV/10^6^ sperm cells/mL). According to the manufacturer’s recommendations, an sORP value ≥ 1.38 was considered abnormal and related to oxidative stress in semen. This means that values of ≤1.37 were normal.

### 2.5. Statistical Analysis

Descriptive statistics were used to define continuous variables (expressed as the median and range, and the mean ± standard deviation). As the variables were not normally distributed (verified using the Shapiro–Wilk test), the nonparametric Kruskal–Wallis test was applied to compare quantitative variables between infertile men with leukocytospermia, infertile men without leukocytospermia, and nonleukocytospermic volunteers with proven fertility. The receiver operating characteristic (ROC) curve and the area under the curve (AUC) were applied to calculate the thresholds of SDF and sORP to discriminate infertile men from fertile individuals in the study population. The ROC analysis took into account the standard error (SE), 95% confidence interval (95% CI), sensitivity, and specificity. The AUCs were as follows: 0.9–1.0 excellent predictive value, >0.8–0.9 good predictive value, >0.7–0.8 satisfactory predictive value, >0.6–0.7 moderate predictive value, and 0.5–0.6 insufficient predictive value. Based on the ROC analysis, the percentages of subjects with SDF and sORP above the estimated thresholds were calculated, and their distributions (prevalences) in the study groups were verified by the Chi^2^ test. The Spearman rank correlation coefficient (r_s_) was used to describe the relationships between SDF, sORP, and conventional sperm characteristics. To interpret the strength association between the study parameters, the following levels of correlation were presumed: <0.2 lack of linear dependence, 0.2–0.4 weak dependence, 0.4–0.7 moderate dependence, 0.7–0.9 strong dependence, and >0.9 very strong dependence. A *p* value < 0.05 was considered significant for all statistical tests. Data analysis was performed using Statistica version 13.3 (StatSoft, Krakow, Poland) and MedCalc version 18.2.1 (MedCalc Software, Ostend, Belgium).

## 3. Results

### 3.1. Infertile vs. Fertile Men

The infertile men with leukocytospermia were statistically older than fertile men (medians: 34.00 vs. 32.00 years), but infertile men without leukocytospermia did not differ in age from control subjects (Table 2). Infertile subjects, with/without leukocytospermia, had significantly lower standard sperm quality than men with proven fertility. The values of the sperm concentration, total sperm count, sperm morphology, progressive motility, and vitality were lower; in turn, the value of TZI and the numbers of sperm cells with head, midpiece, and tail defects and excess residual cytoplasm were higher in both infertile groups. Additionally, infertile subjects had a higher concentration of round cells of the spermatogenic lineage. Furthermore, nonstandard sperm tests showed that infertile men with/without leukocytospermia had significantly higher SDF (median: 24.00% and 19.00%, respectively), raw ORP (median: 49.80 and 56.50 mV), and sORP (median: 2.05 and 4.90, respectively) than the fertile control group (median for SDF: 13%; median for raw ORP: 35.20 mV; median for sORP: 0.62) (Table 2).

**Figure 1:**
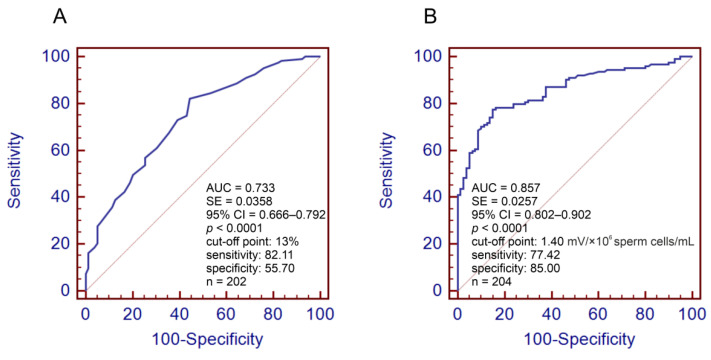
Receiver operating characteristic (ROC) curve and cutoff point for SDF (**A**) and sORP (**B**) to discriminate infertile men from fertile men. 95% CI—95% confidential interval, AUC—area under the curve, SE—standard error, *p*—statistical significance between obtained AUC vs. AUC = 0.500, statistical significance was reached when *p* < 0.05, SDF—sperm DNA fragmentation, sORP—static oxidation-reduction potential. The following levels of AUC were presumed: >0.7–0.8—satisfactory predictive value; >0.8–0.9—good predictive value.

The ROC analysis revealed that the calculated SDF threshold of 13% had a satisfactory predictive value (AUC = 0.733) (Figure 1A) and the threshold of 1.40 sORP had a good predictive value (AUC = 0.857) (Figure 1B) to distinguish infertile men from fertile men. Based on the obtained data, the frequencies of men with >13% SDF and >1.40 sORP and ORs for >13% SDF and >1.40 sORP were calculated. The data showed that there was a significantly higher prevalence of >13% SDF in both infertile groups with and without leukocytospermia (82.61% and 81.81%, respectively) than in fertile individuals (44.30%) (Table 3). These results are in agreement with the OR analysis. The risk for >13% SDF was almost 6-fold higher in both infertile groups than in fertile participants (Table 4). Moreover, significantly more infertile participants with >1.40 sORP were found in the group with/without leukocytospermia (65.95% and 84.42%, respectively) than in the group with proven fertility (16.25%) (Table 3). These findings are consistent with the OR analysis. Infertile men with leukocytospermia had an almost 10-fold higher OR for >1.40 sORP than fertile men, but in infertile men without leukocytospermia, this risk was dramatically elevated by up to 28-fold (Table 4).

### 3.2. Infertile Leukocytospermic Men vs. Infertile Nonleukocytospermic Men

Unexpectedly, there was no significant difference in the basic semen characteristics (with the exception of the round cells of the spermatogenic lineage), SDF, and raw ORP, but there was a surprising difference in sORP between the two infertile groups. Males with leukocytospermia had significantly lower sORP than males without leukocytospermia (median: 2.05 vs. 4.90) (Table 2). These findings were in agreement with the prevalence and OR analysis. There were no differences in the prevalence of >13% SDF between the two infertile groups (Table 3); moreover, the risk for >13% SDF was similar in these groups (Table 4). However, a lower prevalence of >1.40 sORP was found in the infertile group with leukocytospermia than in the nonleukocytospermic group (65.95% vs. 84.42%, respectively) (Table 3). Furthermore, in the infertile group with leukocytospermia, the risk for >1.40 sORP was significantly lower (OR = 0.3577) in relation to infertile men without leukocytospermia (Table 4).

### 3.3. Correlations between Study Parameters

Spearman rank correlation coefficient analysis revealed significant negative linear associations between SDF, raw ORP, or sORP and sperm concentration (r_s_ = −0.2777, r_s_ = −0.2095, and r_s_ = −0.8132, respectively) and total sperm count (r_s_ = −0.2590, r_s_ = −0.2093, and r_s_ = −0.7617, respectively). Whereas SDF or sORP also negatively correlated with sperm morphology (r_s_ = −0.4889 and r_s_ = −0.5392, respectively), sperm progressive motility (r_s_ = −0.5074 and r_s_ = −0.6034, respectively), eosin-negative (live) sperm cells (r_s_ = −0.5044 and r_s_ = −0.4852, respectively), and HOS test-positive (live) sperm cells (r_s_ = −0.4341 and r_s_ = −0.3315, respectively). Additionally, SDF and sORP (but not raw ORP) positively correlated with TZI (r_s_ = 0.2556 and r_s_ = 0.5076, respectively), sperm head defects (r_s_ = 0.4024 and r_s_ = 0.4616, respectively), and sperm midpiece defects (r_s_ = 0.2570 and r_s_ = 0.5232), respectively. Moreover, only sORP negatively correlated with nonprogressive sperm motility (r_s_ = −0.2698) and positively correlated with sperm tail defects (r_s_ = 0.3093) and sperm cells with excess residual cytoplasm (r_s_ = 0.2956). Furthermore, a positive relationship between SDF and raw ORP or sORP was found (r_s_ = 0.2655, r_s_ = 0.3853, respectively). Surprisingly, the concentration of leukocytes positively correlated only with SDF (r_s_ = 0.2647) and not with raw ORP or sORP. Finally, the positive correlation between raw ORP and sORP was confirmed (r_s_ = 0.5545) (Table 5).

## 4. Discussion

### 4.1. Relationships between Male Infertility, SDF, and sORP

The obtained results reveal that both basic semen parameters and the integrity of sperm nuclear DNA are significantly affected in infertile men with/without leukocytospermia (medians: 24% and 19%, respectively; *p* ≥ 0.05), in contrast to fertile men (median: 13%). Moreover, taking into account the differences in raw ORP and sORP between the compared groups, we can assume that OS in the semen of infertile men is much more frequent. Moreover, SDF and sORP correlated negatively (weak, moderate, or strong dependence) with basic sperm parameters (sperm count, morphology, motility, and vitality). Moreover, in our study, in contrast to sORP, raw ORP did not correlate with sperm morphology (including TZI), motility, and vitality. It seems that the results of sORP and conclusions based only on this parameter should be considered carefully. It should be highlighted that the sORP result is strongly influenced by the sperm concentration. The question of how to interpret ORP results was raised by Joao et al. [43]. According to the manufacturer protocol, ORP measured in millivolts (mV) should be adjusted for the sperm concentration and represented as the static ORP (sORP, expressed as mV/10^6^ sperm cells/mL) to show the transfer of electrons between oxidants and reductants [44]. However, Joao et al. [43] questioned the validity of normalization of the results to the mV/10^6^ sperm cells/mL. In the original paper [43], the authors did not show relevant differences in the comparison of men with normozoospermia and abnormal semen parameters when ‘absolute sORP’ (expressed as the raw result in mV) was compared, but when ’sORP index’(expressed as mV/10^6^ sperm cells/mL) was used, the compared groups differed significantly. Additionally, Joao et al. [43] revealed significant negative linear associations only between ‘absolute sORP’ and sperm morphology and the concentration of immature germ cells, but when ‘sORP index’ was used, there were negative correlations with the sperm concertation, total sperm count, total motility, vitality, and morphology, and positive correlations with the semen volume, sperm chromatin decondensation, polymorphonuclear leukocytes, DNA fragmentation, and concentration of immature germ cells. Therefore, the authors [43] concluded that MiOXSYS is related to the oxidation–reduction potential of the seminal fluid (sperm environmental) rather than oxidative stress in sperm and the necessity and justification for the calculation of the ‘sORP index’ is controversial. Therefore, in cases of severe oligoozoospermia, the value of sORP might be overstated. This issue also indicates the need for further meta-analyses to thoroughly compare the clinical utility of raw ORP and sORP.

**Figure 2:**
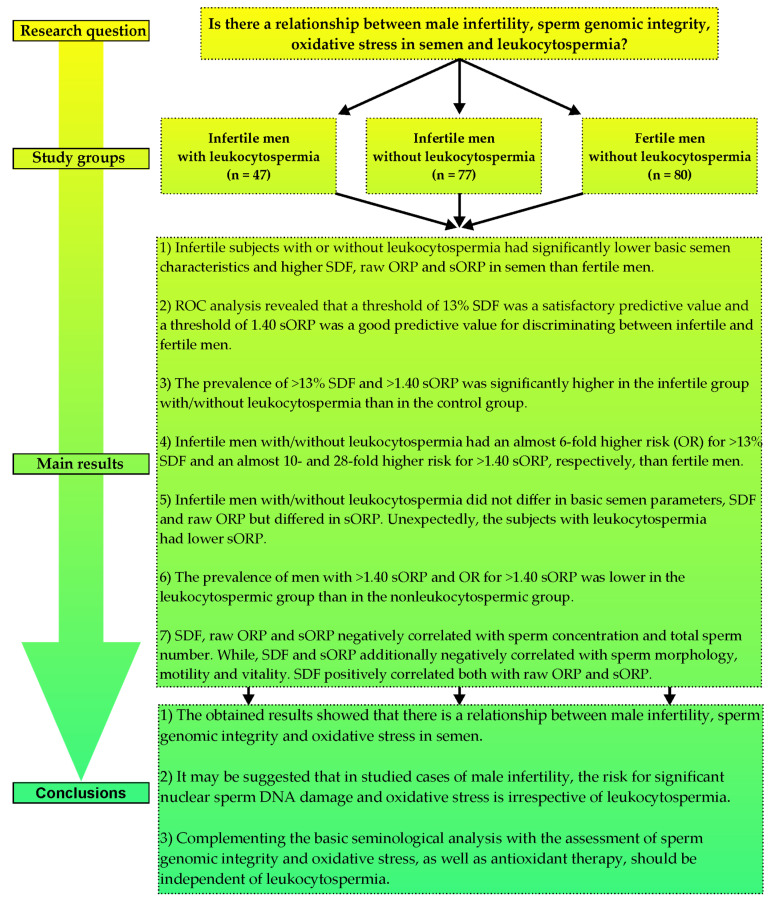
The research strategy. OR—odds ratio, ROC—receiver operating characteristic, SDF—sperm DNA fragmentation, ORP—oxidation-reduction potential (expressed as mV), sORP—static oxidation-reduction potential (expressed as mV/10^6^ sperm cells/mL).

It is worth pointing out that in groups of men struggling with infertility, we noted significantly more abnormalities in the sperm head, midpiece, and tail and significantly more immature sperm cells with excess residual cytoplasm. These observations are important because immature and morphologically abnormal spermatozoa are considered one of the major endogenous sources of ROS, which may result in decreased integrity of the sperm genome [16,19,20,23,45,46,47,48,49]. Furthermore, in our study, sperm DNA fragmentation positively correlated with the oxidation-reduction potential in semen. In other reports, similar findings were presented. The researchers estimated that the sORP values were higher in semen samples with abnormal quality (low number, motility, and/or normal morphology) than in samples with normal basic parameters. Additionally, infertile patients had higher sORP values than fertile donors [40,50]. Moreover, the authors found that in a group of infertile patients, sORP negatively correlated with the total sperm number, motility, and morphology [40,50,51,52,53,54] but positively correlated with sperm DNA fragmentation [40,53]. Considering our findings above and the cited data, we can conclude that infertility coexisting with decreased basic semen parameters and sperm genomic integrity could be caused by oxidative stress in semen (Figure 2).

Our other results confirm the above assumption. Using ROC analysis, we established the cutoff values of SDF and sORP to distinguish infertile men from fertile men. The thresholds of 13% SDF and 1.40 sORP had satisfactory and good predictive values, respectively (see the Materials and Methods). These calculated findings suggest that SDF > 13% and sORP > 1.40 can be associated with an increased risk of infertility. In both infertile groups, the prevalence and the risk (OR) of >13% SDF and >1.40 sORP were significantly higher than those in the fertile group. Up to 82.6% of infertile men had >13% SDF, and the OR for >13% SDF was almost 6-fold higher than that in fertile controls. In turn, up to almost 85% of infertile men had >1.40 sORP in semen, and OR for >1.40 sORP was almost 28-fold higher than that in fertile controls. It should be highlighted that SDF ≤ 15% was considered by other authors as a low level of nuclear DNA damage and was associated with a high fertility potential in men [55,56,57,58,59,60,61]. Similarly, our previous studies revealed that SDF in the range 18–20% had a satisfactory predictive value for distinguishing between healthy volunteers with normozoospermia from men with abnormal sperm parameters, healthy volunteers with normozoospermia from infertile men, and men with normal sperm morphology vs. men with teratozoospermia [37,39,41]. It cannot be omitted that recently published papers indicated that 20% SDF should be considered the optimal cutoff value to discriminate fertile men from infertile men. This cutoff point is common to many tests in use, such as the SCSA, TUNEL, SCD, and comet assay recommended by WHO 2021 for extended examinations of human sperm samples [4,8,62].

Regarding our results, very similar cutoff points of sORP were calculated by Agarwal et al. [54,63] and Arafa et al. [50], who showed that sORP 1.36, 1.42, or 1.41 can differentiate infertile from fertile subjects. Additionally, Cicek et al. [51] confirmed that men with sORP >1.36 had a significantly lower total sperm count, sperm concentration, total motile sperm, progressive motility, and fast forward progressive motility. In conclusion, SDF and sORP, as clinical advanced tests for the assessment of fertility status, have important utility and will help clinicians better diagnose and manage male factor infertility (Figure 2).

### 4.2. Decreased Sperm Genomic Integrity and Oxidative Stress in Semen Can Occur Irrespective of Leukocytospermia

Comparisons between infertile men with leukocytospermia and those without leukocytospermia in our study did not reveal any significant differences in the sperm count, morphology, motility, vitality, or, more importantly, the integrity of sperm nuclear DNA. The risk for significant nuclear sperm DNA damage was similar in both infertile groups. However, basic semen parameters and sperm DNA integrity were significantly decreased compared to the fertile group, which was mentioned in the previous subunit. The obtained SDF values for subjects with/without leukocytospermia suggested that sperm DNA damage was significantly elevated compared to the fertile group irrespective of leukocytospermia. Moreover, in both infertile groups, oxidative stress in semen was shown (medians of sORP were ≥1.38). Unexpectedly, there was no significant difference in raw ORP, but there was in sORP. The sORP value was significantly lower in infertile men with leukocytospermia vs. those without leukocytospermia (median: 2.05 vs. 4.90). As mentioned in the previous subunit, the sORP result depended on the sperm concentration, which in our study was higher (but not statistically) in the leukocytospermic group than nonleukocytospermic. Probably this could have contributed to the lower sORP result noted in the first mentioned group. Additionally, the number of subjects with sORP > 1.40 (calculated cutoff point to discriminate fertile men from infertile men) and the risk for sORP > 1.40 were lower in cases of leukocytospermia. The risk for oxidative stress in semen of men with leukocytospermia was almost 3-fold lower (OR = 0.3577). These findings suggest that decreased sperm genomic integrity and oxidative stress in semen can occur irrespective of leukocytospermia (Figure 2).

Our results and suggestions are partly in agreement with the studies of other authors [64,65]. Alshahrani et al. [64] showed significantly lower SDF in a group of infertile leukocyte-negative men (total absence of leukocytes in semen) vs. infertile men with low-level leukocytes (nonleucytospermic group, leukocyte concentration 0.1–0.9 ×10^6^/mL ejaculate). The authors did not show significant differences in SDF between leukocyte-negative men vs. the infertile leukocytospermic group and between the two leukocyte-positive groups. In turn, Arafa et al. [65] reported that there was an association between OS in semen and sperm quality, including SDF. However, the oxidation-reduction potential in semen was not influenced by leukocytospermia. Liu et al. [13] showed that there were no correlations between the leukocyte concentration, basic semen parameters, sperm cell number, and damaged DNA and 8-OHdG expression in a group of infertile men. Furthermore, some researchers did not indicate that leukocytes significantly influence assisted reproductive technology outcomes [66,67]. Both in original research [66] and meta-analysis [67], the harmful effect of leukocytospermia on in vitro fertilization (IVF) and intracytoplasmic sperm injection (ICSI) was not confirmed. Groups of patients, irrespective of leukocytospermia, did not differ in assessed embryo parameters, the course of pregnancy, or live birth rates.

However, it cannot be omitted that some authors showed a significant impact of leukocytospermia on seminological parameters. The results of the linear regression performed by Eini et al. [24] revealed that there was a moderate correlation between leukocytospermia and sperm DNA fragmentation in a group of infertile men, and the authors concluded that bacterial infection significantly correlated with leukocytospermia could impair the male fertility potential by decreasing basic semen parameters and sperm genomic integrity. Similarly, numerous authors [25,28,68,69,70] have proved that leukocytospermia contributes to a significant decrease in the sperm nuclear DNA integrity. Moreover, Pratap et al. [25] showed that the level of adenosine deaminase (ADA—an enzyme released mainly by lymphocytes and macrophages) in semen correlated positively with SDF.

Taking into account the fact that leukocytospermia is often considered one of the major factors of oxidative stress in semen and consequently contributes to male infertility [10,12,23,25,28,68,69,70], our research provides important novel data: leukocytospermia is not always the main source of extensive generation of ROS in the semen of infertile patients. In our opinion, male infertility is related to an abnormal oxidation-reduction potential in semen with high probability, but the presence or absence of leukocytospermia should not be the only criterion for verification of oxidative stress and implementation of antioxidant therapy (Figure 2).

Regarding our findings, it is interesting to consider why the sORP value was higher in the group of infertile leukocytospermia-negative men vs. men with leukocytospermia. It is possible that the subjects without high levels of inflammatory cells were more exposed independent of leukocytospermia risk factors for extensive ROS generation. It should be noted that there are many sources of oxidative stress in the male reproductive tract, as described in the Introduction. Briefly, endogenous (e.g., varicocele, immature sperm cells) and exogenous (e.g., smoking, alcohol, pollution, radiation, obesity) risk factors for oxidative stress are known [6,9,18,19,20,21,27]. Furthermore, we can assume that in the semen of infertile men without leukocytospermia, total antioxidant capacity (TAC), antioxidant enzymes, and molecule activity are decreased. An association between male infertility, the level of TAC, and the activity of antioxidant enzymes and molecules (catalase, superoxide dismutase, glutathione) was confirmed [10,46,71,72,73,74,75,76].

## 5. Conclusions

The obtained results showed that there is a relationship between male infertility, sperm genomic integrity, and oxidative stress in semen. Compared to fertile men, infertile men had a higher risk for significant nuclear sperm DNA damage and oxidative stress irrespective of leukocytospermia. It may be suggested that in the study cases of male infertility, leukocytospermia was not necessarily the only cause of oxidative stress because nonleukocytospermic infertile men had a higher risk for oxidative stress than leukocytospermic subjects. In turn, the risk for significant nuclear sperm DNA damage was similar in both infertile groups but significantly higher than that in fertile controls. These findings indicated that sperm DNA damage and oxidative stress occurred irrespective of leukocytospermia. Hence, the assessment of oxidative stress, apart from the evaluation of sperm DNA integrity, should complement the standard seminological analysis, and antioxidant therapy should be independent of leukocytospermia. It should be highlighted that the impact of leukocytospermia on male fertility is still an open question, and it is justified to conduct research to expand knowledge in this area (Figure 2).

## 6. Limitations of this Study

The results described in our paper indicate the existence of certain associations between male fertility status, the integrity of sperm nuclear DNA, oxidation-reduction potential in semen, and leukocytospermia. However, we are fully aware that there is a need for research conducted on a larger group of patients because cohort studies would probably enable the provision of more reliable statistical data. Furthermore, the control group was composed of healthy fertile men. We cannot be absolutely sure that there were no changes in their bodies and reproductive systems between the time of conception and the day of the semen analysis that could affect the verified parameters. Although, the participants were asked about the possibility of exposure to some new harmful factors (e.g., severe diseases, testicular injury, exposure to dangerous environmental factors, changes in lifestyle or work). It might seem that, in an ideal experimental model, semen samples should be collected and analyzed within 3 months after successful conception (time of spermatogenesis with the sperm maturation cycle in epididymis), but early pregnancy cannot be synonymous with live birth.

It should also be noted that the methods used in our study have some advantages and disadvantages. Sperm DNA fragmentation was evaluated by the diagnostic and standardized test SCD, one of a few tests recommended by the most recent 6th edition of the WHO manual for assessment of human semen [62]. However, it should be noted that this is not a flawless method. The strengths and limitations of the SCD test were presented and discussed in our pervious papers [37,39,42].

MiOXSYS is one of three useful and clinically available measurements of ROS in semen recommended by the WHO [62]. Although it is a relatively novel method, it has been extensively discussed in scientific publications [44]. The most important advantages of MiOXSYS are rapid result, high sensitivity, small sample volume required for analysis (only 30 µL), good reproducibility, long time for analysis (the results can be obtained up to 2 h post ejaculation), fresh and frozen samples can be used, and the measurements can be performed in raw semen and seminal fluid. Finally, the methodology is very simple and standardized as the manufacturer provides a ready-to use protocol [44,62]. On the other hand, there are known disadvantages of this method. MiOXSYS cannot differentiate different types of ROS radicals, the results are influenced by a low sperm count (especially severe oligozoospermia), and the method is temperature-sensitive. Moreover, the sensors required for testing are quite expensive [44].

## Figures and Tables

**Table 1:** Sperm classification in the sperm chromatin dispersion test.

Sperm Category	Phenotype of Sperm Cells Head	Description
Sperm cells with non-fragmented nuclear DNA	Head with big halo	Halo equal to or higher than the diameter of the core of spermatozoa
	Head with medium halo	Halo > 1/3 of the diameter of the core of spermatozoa
	Head with small halo	Halo ≤ 1/3 of the diameter of the core of spermatozoa
Sperm cells with fragmented nuclear DNA	Head without halo	Absence of halo but strongly stained core
	Head with no halo and degraded chromatin	Absence of halo and simultaneously an irregularly or weakly stained core

**Table 2:** Descriptive statistics and comparisons of standard semen parameters, SDF, and sORP between the study groups.

Parameters	Total (n = 204)	Group 1: Infertile Men with Leukocytospermia (n = 47)	Group 2: Infertile Men without Leukocytospermia (n = 77)	Group 3: Fertile Men without Leukocytospermia (n = 80)	*p* 1 vs. 3	*p* 2 vs. 3	*p* 1 vs. 2
Median (Range) Mean ± SD							
Age (y)	33.00 (22.00–49.00) 33.00 ± 4.80	34.00 (27.00–46.00) 34.17 ± 4.37	33.00 (25.00–49.00) 33.55 ± 4.95	32.00 (22.00–47.00) 31.78 ± 4.68	0.015445	NS	NS
Semen volume (mL)	3.00 (0.50–8.00) 3.21 ± 1.59	3.00 (0.50–8.00) 3.20 ± 1.79	3.00 (0.50–6.50) 3.04 ± 1.41	3.00 (0.50–8.00) 3.37 ± 1.64	NS	NS	NS
Sperm concentration (×10^6^/mL)	23.08 (0.05–210.00) 34.62 ± 37.12	16.75 (0.44–130.00) 25.15 ± 26.27	7.00 (0.05–146.50) 18.01 ± 25.47	50.50 (4.80–210.00) 56.16 ± 41.50	0.000008	<0.000001	NS
Total number of spermatozoa (×10^6^)	63.91 (0.25–840.00) 108.68 ± 137.68	52.25 (0.50–575.00) 74.22 ± 91.04	21.30 (0.25–365.75) 50.25 ± 68.86	139.41 (21.60–840.00) 185.16 ± 171.27	0.000008	<0.000001	NS
Morphologically normal spermatozoa (%)	2.00 (0.00–13.00) 2.47 ± 3.07	0.00 (0.00–10.00) 1.38 ± 2.38	0.00 (0.00–9.00) 1.12 ± 1.77	4.00 (0.00–13.00) 4.41 ± 3.42	<0.000001	<0.000001	NS
TZI	1.75 (1.15–2.58) 1.78 ± 0.29	1.81 (1.46–2.58) 1.84 ± 0.28	1.89 (1.36–2.50) 1.90 ± 0.30	1.64 (1.15–2.25) 1.62 ± 0.22	0.000391	<0.000001	NS
Total sperm head defects (%)	96.00 (72.00–100.00) 94.75 ± 5.13	98.00 (72.00–100.00) 95.93 ± 5.71	98.00 (87.00–100.00) 94.81 ± 3.71	92.00 (92.00–100.00) 94.75 ± 5.13	0.000005	< 0.000001	NS
Total sperm midpieces defects (%)	41.50 (9.00–84.00) 42.74 ± 5.13	42.00 (20.00–78.00) 43.80 ± 15.06	51.00 (9.00–84.00) 50.10 ± 17.18	36.50 (9.00–73.00) 35.03 ± 13.75	0.020289	< 0.000001	NS
Total sperm tail defects (%)	29.00 (6.00–88.00) 31.91 ± 14.90	30.00 (6.00–88.00) 34.36 ± 17.56	37.00 (8.00–65.00) 36.32 ± 15.02	25.00 (6.00–50.00) 26.23 ± 10.92	0.030531	0.000075	NS
Immature sperm with excess residual cytoplasm (%)	n = 203 4.00 (0.00–60.00) 4.99 ± 6.36	n = 46 6.50 (0.00–41.00) 7.21 ± 6.43	4.00 (0.00–60.00) 6.16 ± 8.10	2.00 (0.00–8.00) 2.57 ± 2.62	<0.000001	0.000281	NS
Progressive motility (%)	51.00 (0.00–90.00) 46.91 ± 23.14	39.00 (0.00–74.00) 39.25 ± 22.17	33.00 (0.00–79.00) 34.46 ± 21.03	67.00 (22.00–90.00) 63.40 ± 14.25	<0.000001	<0.000001	NS
Nonprogressive motility (%)	6.00 (0.00–33.00) 7.38 ± 6.42	6.00 (0.00–16.00) 6.10 ± 3.80	5.00 (0.00–16.00) 5.24 ± 3.64	8.00 (0.00–33.00) 10.20 ± 8.45	NS	0.001066	NS
Eosin-negative spermatozoa—live cells (%)	77.00 (3.00–96.00) 75.15 ± 13.56	74.00 (48.00–90.00) 73.25 ± 10.34	74.00 (3.00–96.00) 69.16 ± 16.46	84.00 (62.00–95.00) 82.02 ± 8.06	0.000021	<0.000001	NS
HOS test-positive spermatozoa—live cells (%)	n = 168 77.00 (13.00–95.00) 74.57 ± 11.65	n = 38 73.00 (34.00–92.00) 73.34 ± 73.34	n = 55 71.00 (13.00–90.00) 90.00 ± 69.83	n = 75 80.00 (58.00–95.00) 95.00 ± 78.68	0.034308	0.000117	NS
Peroxidase-positive cells (×10^6^/mL)	0.30 (0.00–27.00) 0.95 ± 2.42	1.75 (1.00–27.00) 3.20 ± 4.37	0.25 (0.00–0.96) 0.32 ± 0.24	0.25 (0.00–0.75) 0.23 ± 0.19	<0.000001	NS	0.0100
Round sperm cells (×10^6^/mL)	n = 203 4.00 (0.00–60.00) 4.99 ± 6.36	n = 46 6.50 (0.00–41.00) 7.21 ± 6.43	4.00 (0.00–60.00) 6.16 ± 8.10	2.00 (0.00–8.00) 2.57 ± 2.62	<0.000001	0.042878	0.008361
SDF (%)	n = 202 17.00 (3.00–48.00) 18.68 ± 8.89	n = 46 24.00 (5.00–44.00) 22.10 ± 8.98	19.00 (7.00–48.00) 21.05 ± 8.77	n = 79 13.00 (3.00–34.00) 14.37 ± 7.16	0.034308	0.000117	NS
Raw ORP (mV)	45.80 (2.10–184.10) 49.26 ± 29.63	49.80 (3.70–175.40) 55.50 ± 37.34	56.50 (14.09–121.80) 55.72 ± 26.23	35.20 (2.10–184.10) 39.36 ± 26.23	0.021902	0.00010	NS
sORP (mV/10^6^ sperm cells/mL)	1.62 (0.02–196.50) 13.22 ± 35.89	2.05 (0.09–128.00) 7.12 ± 19.27	4.90 (0.28–196.50) 29.75 ± 52.49	0.62 (0.02–5.00) 0.91 ± 0.90	0.000007	<0.000001	0.004418

HOS test—hypoosmotic swelling test, n—number of subjects, SD—standard deviation, SDF—sperm DNA fragmentation, ORP—oxidation–reduction potential, sORP—static oxidation–reduction potential, TZI—teratozoospermia index, statistical significance in the Kruskal–Wallis test was reached when *p* < 0.05, NS—not significant.

**Table 3:** Prevalence of SDF and sORP in the study groups.

Calculated SDF and sORP Threshold	Total %(n)	Group 1: Infertile Men with Leukocytospermia %(n)	Group 2: Infertile Men without Leukocytospermia %(n)	Group 3: Fertile Men without Leukocytospermia %(n)	*p* 1 vs. 3	*p* 2 vs. 3	*p* 1 vs. 2
SDF > 13%	n = 202 67.33 (136)	n = 46 82.61 (38)	n = 77 81.81 (63)	n = 79 44.30 (35)	<0.0001	<0.0001	NS
sORP > 1.40 mV/10^6^ sperm cells/mL	n = 204 53.43 (109)	n = 47 65.95 (31)	n = 77 84.42 (65)	n = 80 16.25 (13)	<0.0001	<0.0001	0.0253

n—number of subjects, NS—not significant, SDF—sperm DNA fragmentation, sORP—static oxidation–reduction potential. Statistical significance in Chi^2^ test was reached when *p* < 0.05.

**Table 4:** Odds ratios for SDF and sORP in the study groups.

Calculated SDF and sORP Threshold	Total %(n)	Group 1: Infertile Men with Leukocytospermia %(n)	Group 2: Infertile Men without Leukocytospermia %(n)	Group 3: Fertile Men without Leukocytospermia %(n)	OR1 (95% CI) *p*	OR2 (95% CI) *p*	OR3 (95% CI) *p*
SDF > 13%	n = 202 67.33 (136)	n = 46 82.61 (38)	n = 77 81.81 (63)	n = 79 44.30 (35)	5.9714 (2.4713–14.4289) 0.0001	5.6571 (2.7271–11.7354) <0.0001	0.9474 (0.3637–2.4679) NS
sORP > 1.40 mV/10^6^ sperm cells/mL	n = 204 53.43 (109)	n = 47 65.95 (31)	n = 77 84.42 (65)	n = 80 16.25 (13)	9.9856 (4.2822–23.2853) <0.0001	27.9167 (11.8652–65.6830) <0.0001	0.3577 (0.1510–0.8471) 0.0194

n—number of subjects, NS—not significant, OR—odds ratio, SDF—sperm DNA fragmentation, sORP—static oxidation–reduction potential. OR1—OR for SDF and sORP in infertile men with leukocytospermia vs. fertile men without leukocytospermia; OR2—OR for SDF and sORP in infertile men without leukocytospermia vs. fertile men without leukocytospermia; OR3—OR for SDF and sORP in infertile men with leukocytospermia vs. infertile men without leukocytospermia. Statistical significance in odds ratio test was reached when *p* < 0.05.

**Table 5:** Spearman rank correlation (r_s_) of SDF (n = 202) and sORP (n = 204) with male age and standard semen parameters in the total group.

Parameters	SDF (%) r_s_(*p*)	Raw ORP (mV) r_s_(*p*)	sORP (mV/10^6^ Sperm Cells/mL) r_s_(*p*)
Age (y)	0.1833 (0.009004)	0.0547 (NS)	0.0619 (NS)
Semen volume (mL)	0.0246 (NS)	0.0333 (NS)	0.0170 (NS)
Sperm concentration (×10^6^/mL)	−0.2777 (0.000063)	−0.2095 (0.002625)	−0.8132 (<0.000001)
Total number of spermatozoa (×10^6^)	−0.2590 (0.000198)	−0.2093 (0.002625)	−0.7617 (<0.000001)
Morphologically normal spermatozoa (%)	−0.4889 (<0.000001)	−0.1593 (0.022791)	−0.5392 (<0.000001)
TZI	0.2556 (0.000241)	0.1087 (NS)	0.5076 (<0.000001)
Total sperm head defects (%)	0.4024 (<0.0000001)	0.1070 (NS)	0.4616 (<0.0000001)
Total sperm midpieces defects (%)	0.2570 (0.0002210)	0.1627 (0.020021)	0.5232 (<0.0000001)
Total sperm tail defects (%)	0.1576 (0.0250438)	0.0016 (NS)	0.3093 (0.0000067)
Immature sperm with excess residual cytoplasm (%)	201 0.1959 (0.0053135)	203 0.1110 (NS)	203 0.2956 (0.0000184)
Progressive motility (%)	−0.5074 (<0.000001)	−0.1816 (0.009319)	−0.6034 (0.000001)
Nonprogressive motility (%)	−0.0244 (NS)	−0.1652 (0.018199)	−0.2698 (0.000095)
Eosin-negative spermatozoa—live cells (%)	−0.5044 (<0.000001)	−0.1387 (0.047812	−0.4852 (<0.000001)
HOS test-positive spermatozoa—live cells (%)	n = 166 −0.4341 (<0.000001)	−0.1099 (NS)	n = 168 −0.3315 (0.001457)
Peroxidase-positive cells (×10^6^/mL)	0.2647 (0.000141)	0.1050 (NS)	0.1097 (NS)
Round sperm cells (×10^6^/mL)	0.1238 (NS)	0.0664 (NS)	0.0638 (NS)
SDF (%)	–	0.2655 (0.000133)	0.3853 (<0.000001)
Raw ORP (mV)		–	0.5545 (<0.000001)

HOS test—hypoosmotic swelling test, n—number of subjects, NS—non statistically significant, ORP—oxidation-reduction potential, sORP—static oxidation–reduction potential, SDF—sperm DNA fragmentation, TZI—teratozoospermia index. Statistical significance in rank Spearman correlation was reached when *p* < 0.05. The interpretation of r_s_ value: <0.2 lack of linear dependence. ≥0.2–0.4—weak dependence. >0.4–0.7—moderate dependence. >0.7–0.9—strong dependence. >0.9—very strong dependence.

## Data Availability

The data presented in this study are available on request from the corresponding author.
